# Supplementary material for: Electrosynthesis of 1,4-bis(diphenylphosphanyl) tetrasulfide via sulfur radical addition as cathode material for rechargeable lithium battery
Source: Nat Commun. 2021 May 28;12:3220. doi: 10.1038/s41467-021-23521-1 (PMC8163837; doi:10.1038/s41467-021-23521-1)
Supplement: Supplementary file 1 — Supplementary Information [file 41467_2021_23521_MOESM1_ESM.pdf]

Supplementary information

Electrosynthesis of 1,4-bis(diphenylphosphanyl) tetrasulfide via sulfur radical addition as cathode material for rechargeable lithium battery

Dan-Yang Wang[1,†], Yubing Si[1,†], Wei Guo[1], and Yongzhu Fu[1]*

[1]College of Chemistry, Zhengzhou University, Zhengzhou 450001, P. R. China

[†]These authors contributed equally: Dan-Yang Wang, Yubing Si

*Corresponding author: yfu@zzu.edu.cn (Y. Fu)

Supplementary Method:

Commercial electrolyte 1.0 M lithium bis(trifluoromethanesulfonyl)imide (LiTFSI) in 1,3-dioxolane (DOL):1,2-dimethoxyethane (DME) =1:1 v/v with 0.15 M $LiNO_3$, diphenyl dithiophosphinic acid (DPDTP, 99%), and diethyl dithiophosphate (DEDTP, 99%) were purchased from Alfa Aesar and used as received.

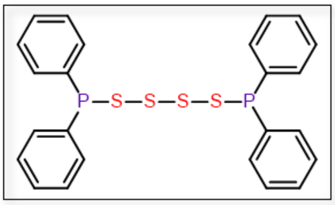

1,4-bis(diphenylphosphanyl)tetrasulfide (BDPPTS), $\mathbf{C_{24}H_{20}P_2S_4}$ 497.99 g mol^{-1} (Pale yellow oily liquid), **^{1}H NMR** (600 MHz, $CDCl_3$, Me_4Si): δ (ppm) 7.91-7.72 (m, 8H), 7.53-7.33 (m, 12H). **^{13}C NMR** (150 MHz, $CDCl_3$, Me_4Si) δ (ppm) 132.97 132.18 131.75 128.41. **^{31}P NMR** (262 MHz, $CDCl_3$, Me_4Si) 69.5. **HRMS** (APCI) m/z: $[M + H]^+$ Calculated for $C_{24}H_{20}P_2S_4$ 498.9996; Found 498.9989.

Supplementary Figures:

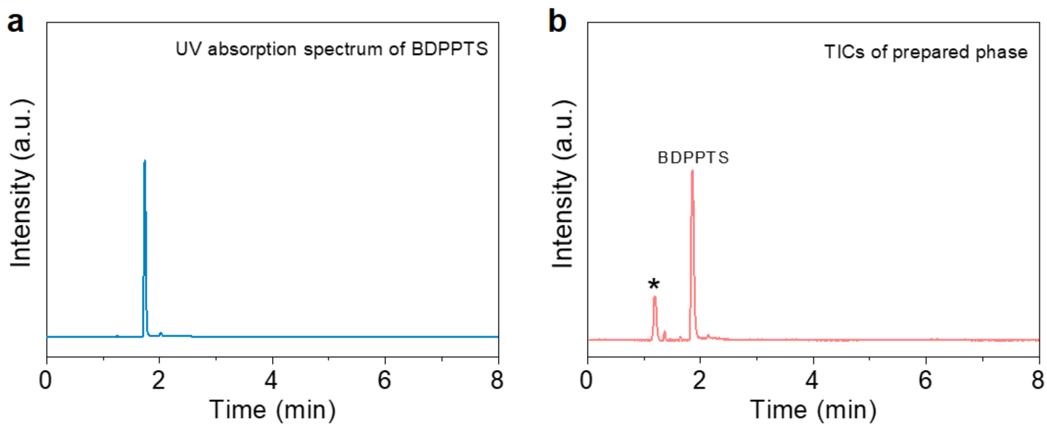

Supplementary Figure 1. The yield is 96% obtained by integrating the peak area in the UV spectrum (a) and the peak BDPPTS of UPLC/MS (b), * represents the content of solvent.

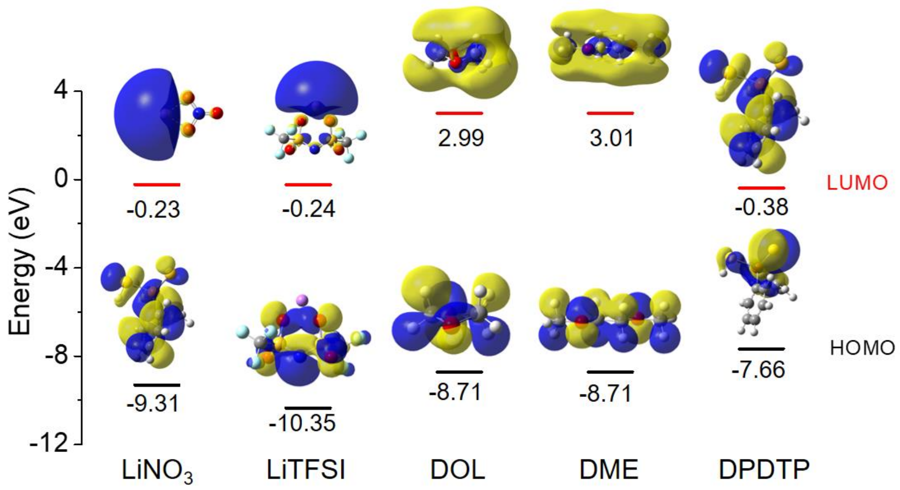

Supplementary Figure 2. The frontier molecular orbital energies and electron density of the lowest unoccupied molecular orbital (LUMO) and highest occupied molecular orbital (HOMO) of the $LiNO_3$, LiTFSI, DOL, DME, and DPDTP as calculated at the PBE0/SMD/cc-pVTZ level.

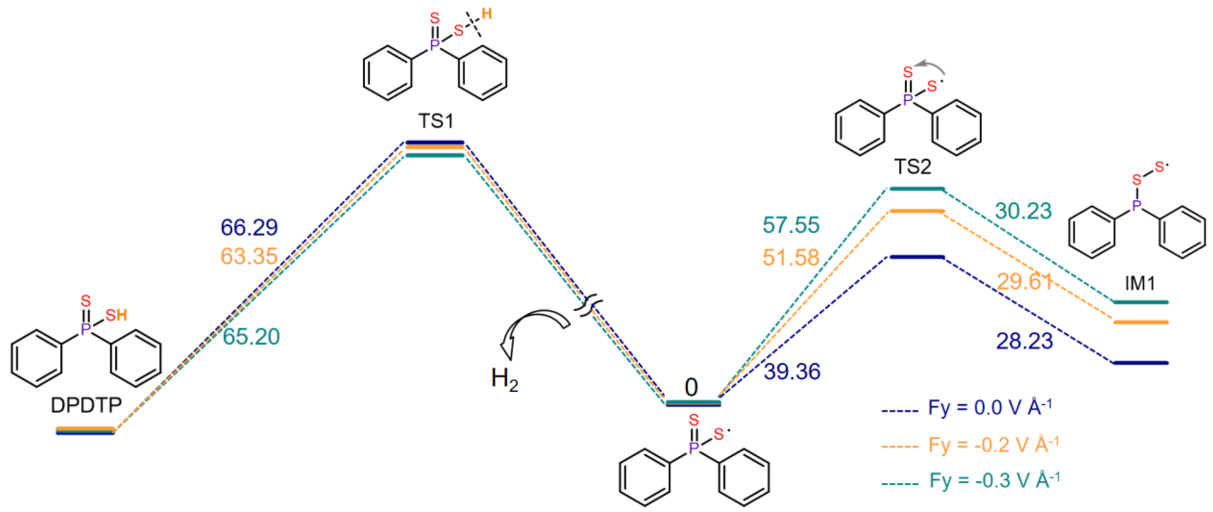

Supplementary Figure 3. Energy profiles (in kcal/mol) for the electrochemical oxidation of DPDTP leading to the formation of linear organopolysulfides BDPPTS (Fy = 0.0 V $Å^{-1}$, dark blue lines; Fy = -0.2 V $Å^{-1}$, yellow lines; Fy = -0.3 V $Å^{-1}$, cyan lines). Because there is no consensus on the actual field strength in the systems, the widely-used field strength of 0.2 and 0.3 V $Å^{-1}$ (Fy + 40 and Fy + 60) were employed to simulated the finite external electric field. Besides, it is well known that the external electric field is a vector quantity, thus the opposite field direction would hinder the hydrogen elimination and sulfur migration. As shown in this figure, the higher energy barriers were observed at the moment of Fy – 40 and Fy – 60. However, it is believed that, in explicit solvent environment, the external electric field would make the solvent molecular more ordered, accelerating the reactions (Ref 13 of the manuscript).

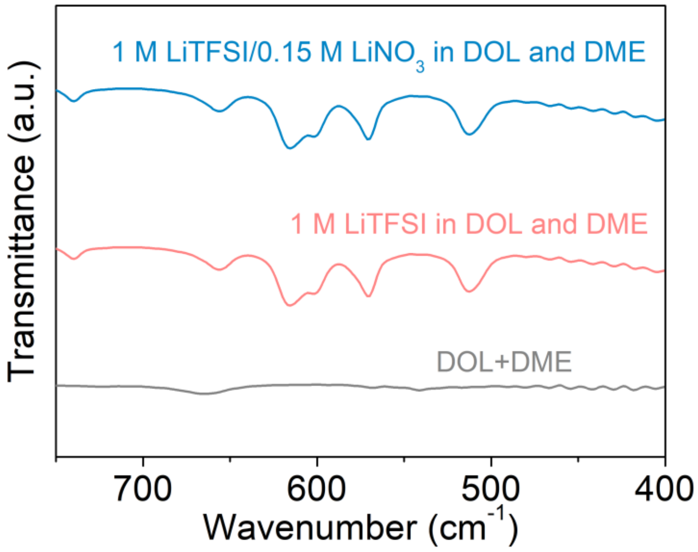

Supplementary Figure 4. FTIR spectra of different electrolyte contents.

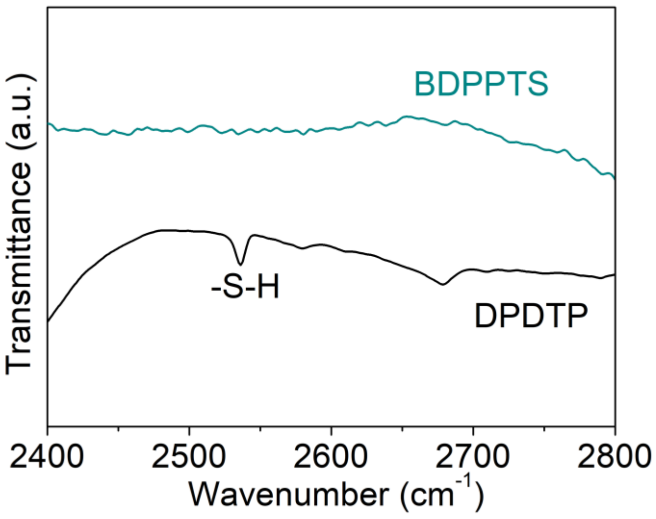

Supplementary Figure 5. FTIR spectra of BDPPTS and DPDTP.

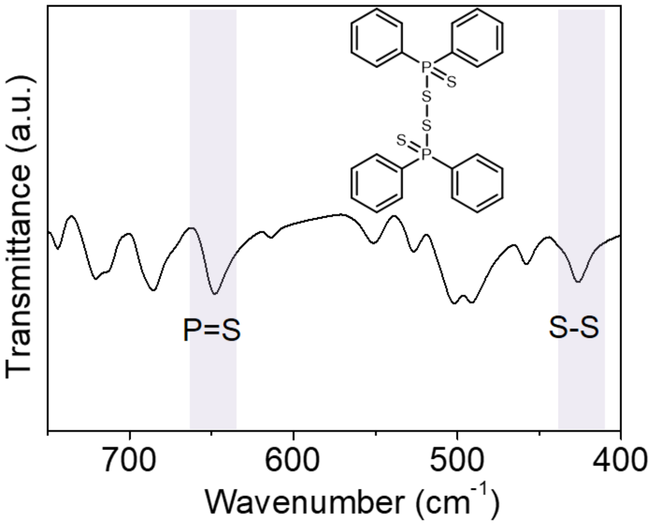

Supplementary Figure 6. FTIR spectrum of the synthesized bis(diphenylphosphinothioyl) disulfide (DPTDS) showing similar peaks to DPDTP except for the S-S peak at 420 cm^{-1}.

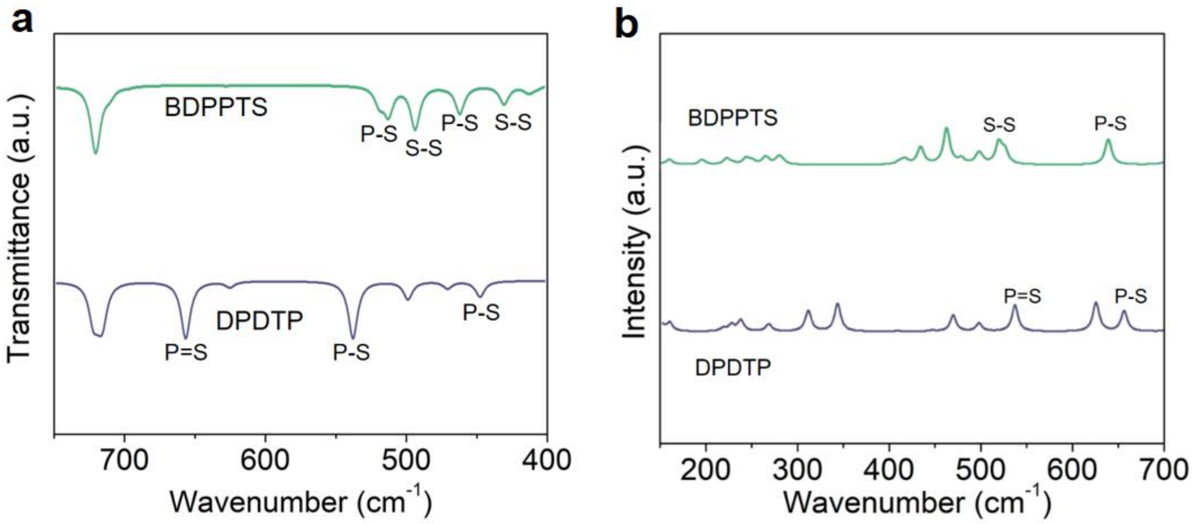

Supplementary Figure 7. Simulated FTIR (a) and Raman (b) spectra of BDPPTS and DPDTP.

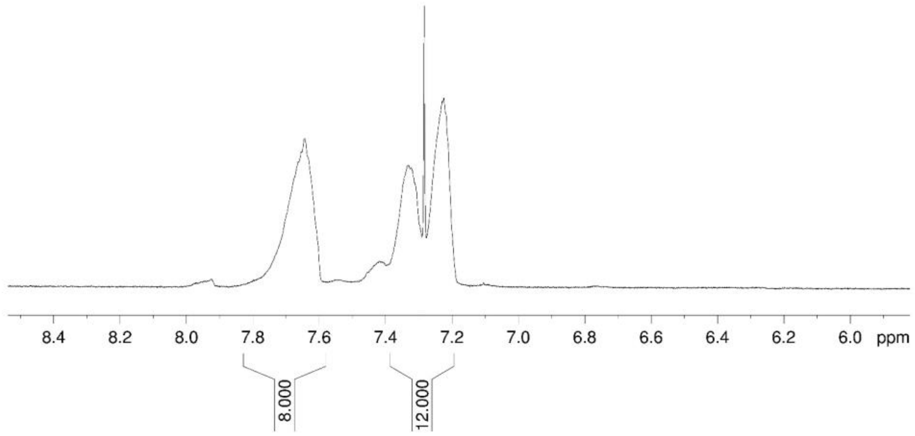

Supplementary Figure 8. ^{1}H NMR spectrum of the BDPPTS.

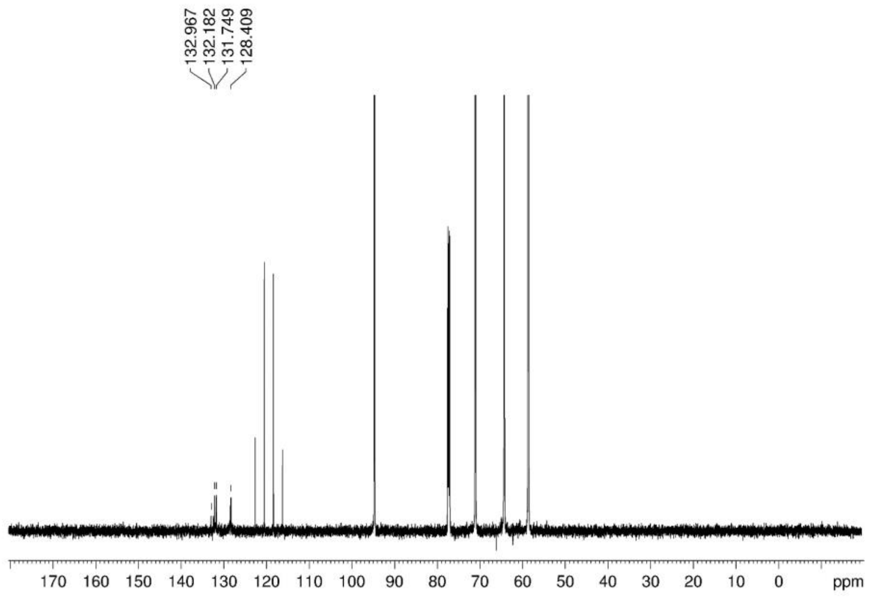

Supplementary Figure 9. ^{13}C NMR spectrum of the BDPPTS.

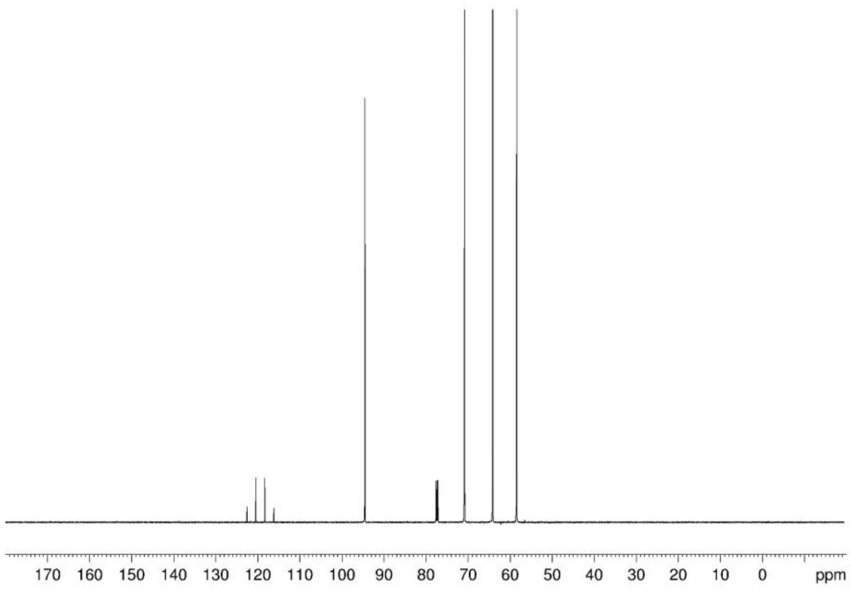

Supplementary Figure 10. ^{13}C NMR spectrum of the solvent as the control test.

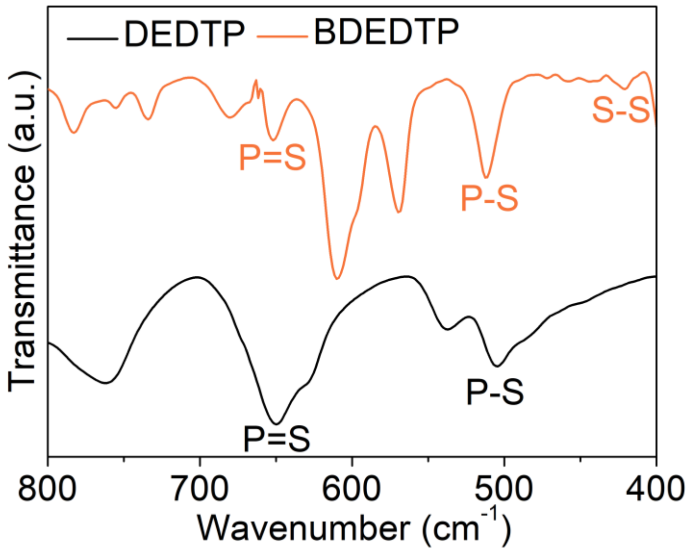

Supplementary Figure 11. FTIR spectra of DEDTP and BDEDTP, retention of P=S double bond and P-S single bond and emergence of S-S bond indicate the formation of dimeric DEDTP.

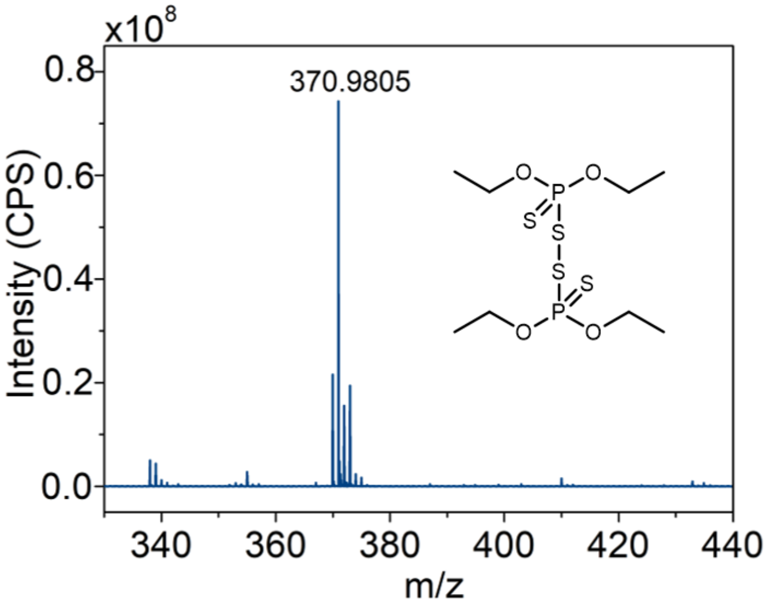

Supplementary Figure 12. The mass spectrum corresponding to the electrooxidation product of BDEDTP that is consistent with above mentioned FTIR analysis result.

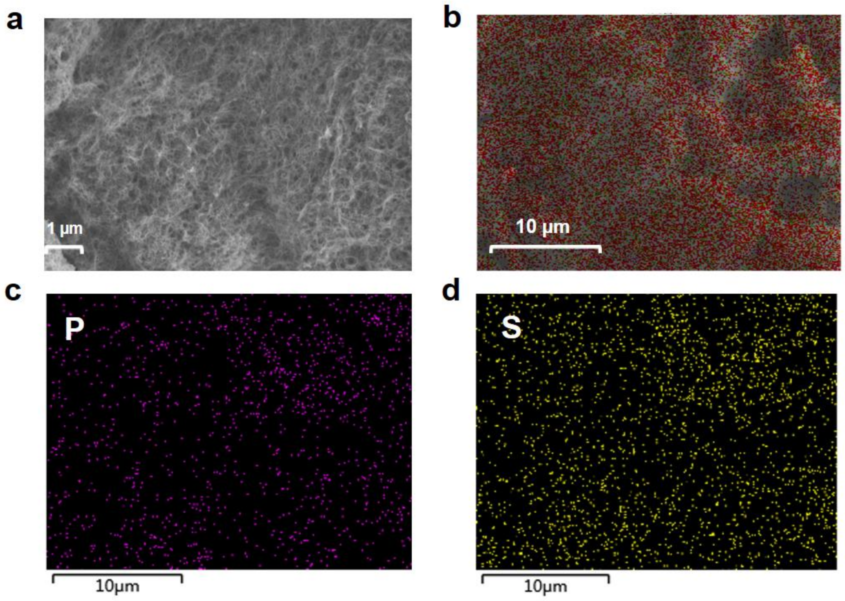

Supplementary Figure 13. SEM image (a) and EDS elemental maps of the prepared cathode (b), (c), and (d).

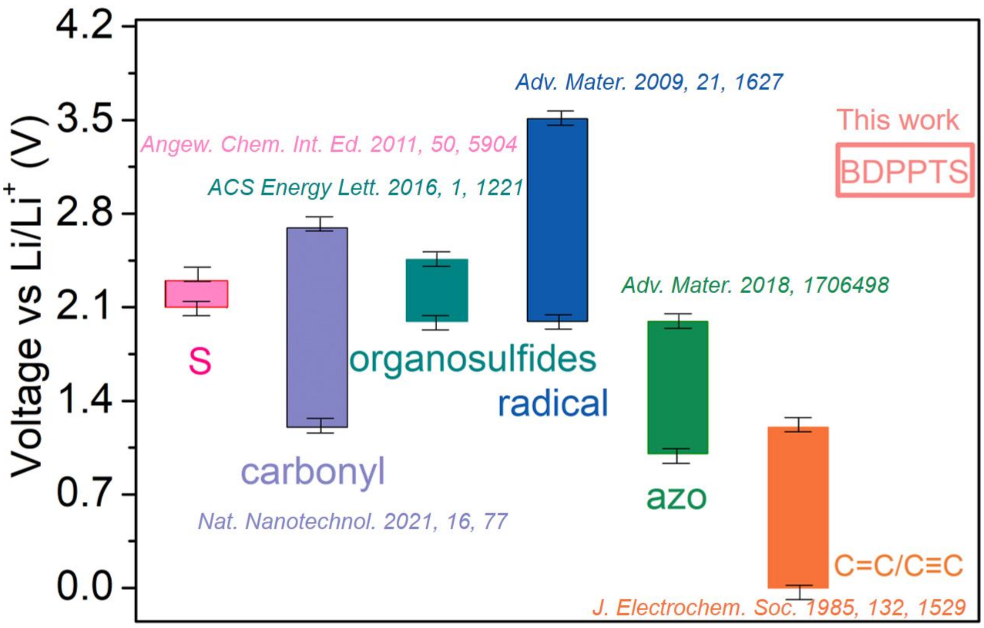

Supplementary Figure 14. Discharge voltage interval summary of sulfur (S), common organic electrodes, and BDPPTS[1-6].

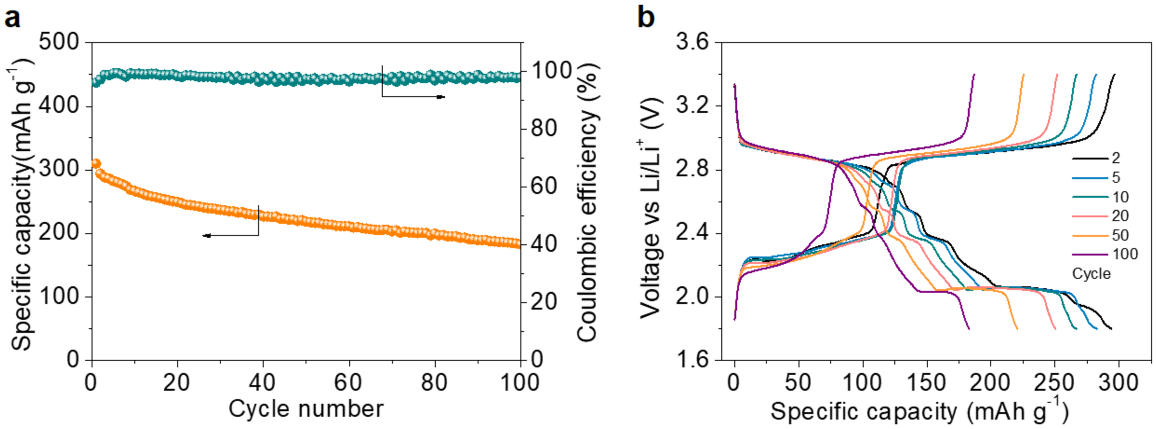

Supplementary Figure 15. (a) Cycling performance of the Li/BDPPTS cell at C/10 rate, (b) Selected charge-discharge voltage profiles.

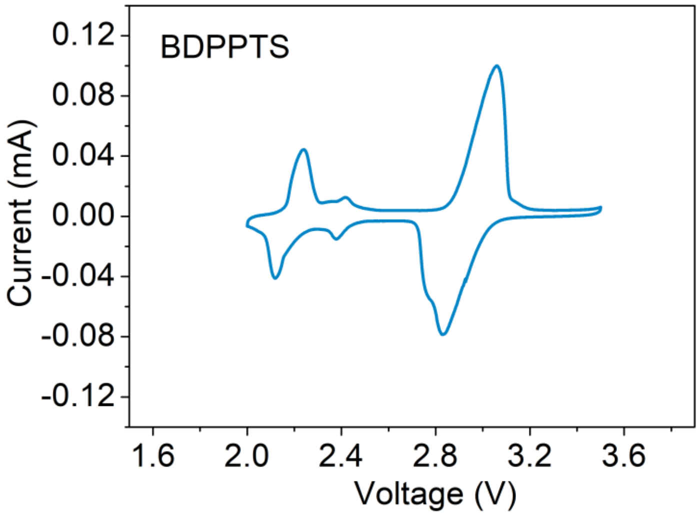

Supplementary Figure 16. Cyclic voltammogram of the Li/BDPPTS cell performed at a scan rate of 0.05 mV s^{-1}.

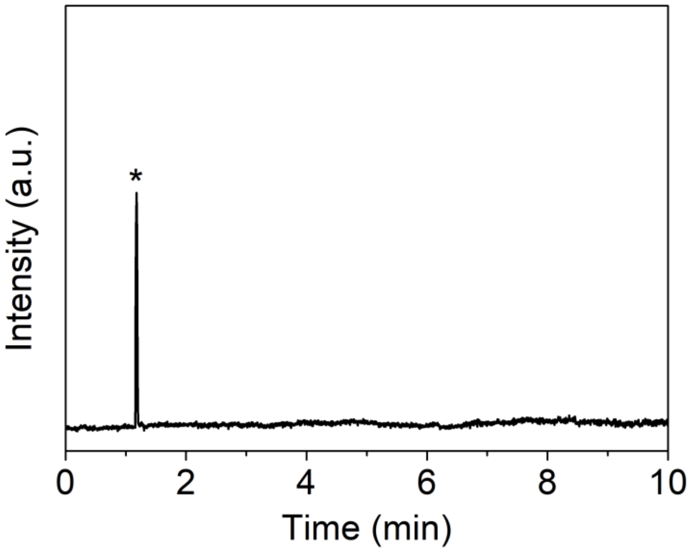

Supplementary Figure 17. Total ion chromatogram (TIC) of the electrolyte.

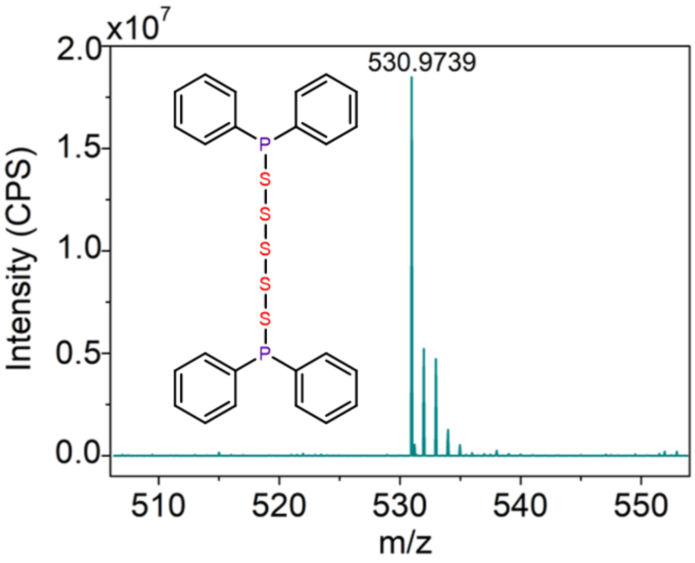

Supplementary Figure 18. The mass spectrum corresponding to the electrooxidation product BDPPPS.

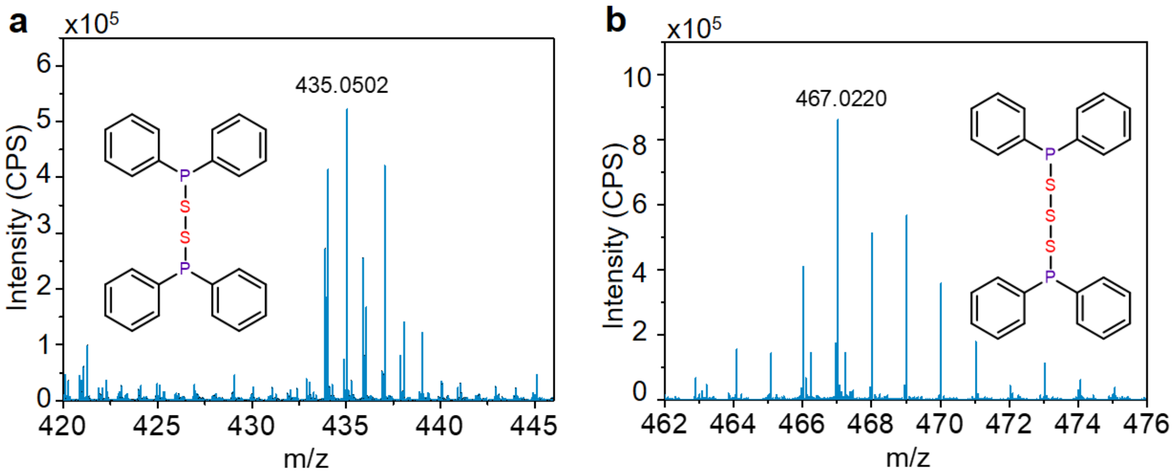

Supplementary Figure 19. The mass spectra corresponding to the electrooxidation product: bis(diphenylphosphanyl)disulfide (a), bis(diphenylphosphanyl)trisulfide (b).

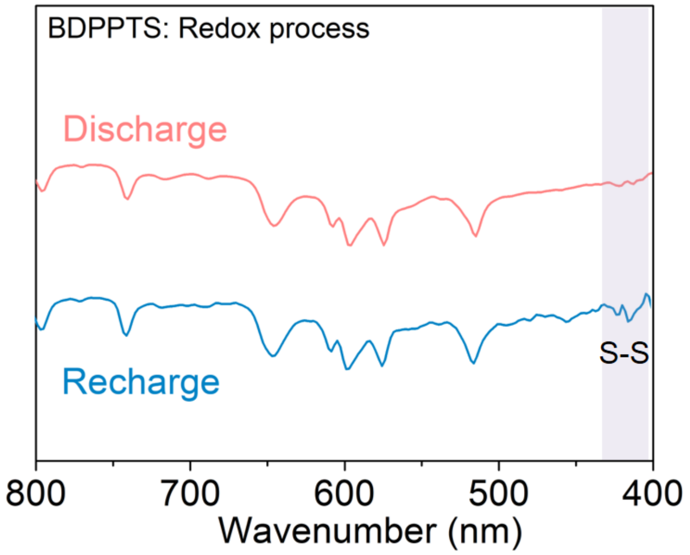

Supplementary Figure 20. FTIR spectra of the discharged and recharged products in the Li/BDPPTS cell.

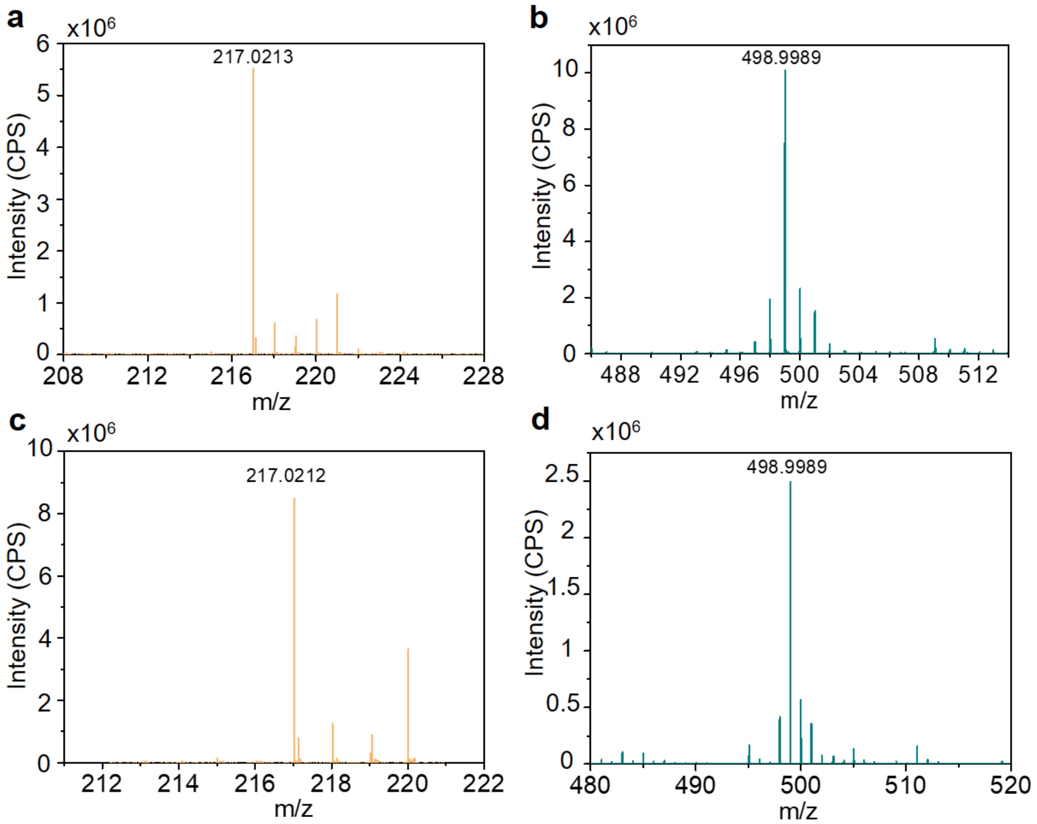

Supplementary Figure 21. The mass spectra of the discharged (a) and recharged (b) products after 10 cycles and the discharged (c) and recharged (d) products in the BDPPTS electrodes after 50 cycles in the Li/BDPPTS cell in the voltage range of 1.8-3.4 V.

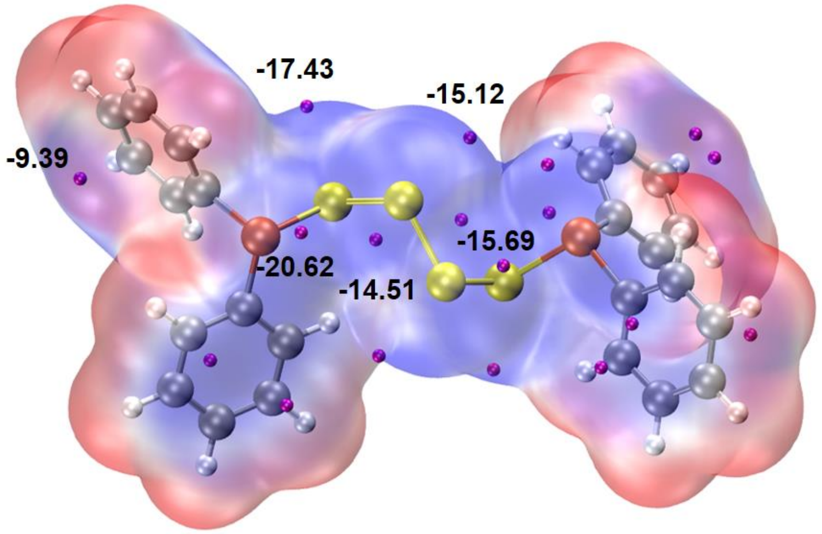

Supplementary Figure 22. The isocontour surfaces of electrostatic potential (ESP) for BDPPTS. The selected local maximum points calculated by Multiwfn program were labeled and illusminated in purple dots (in kcal mol^{-1}).

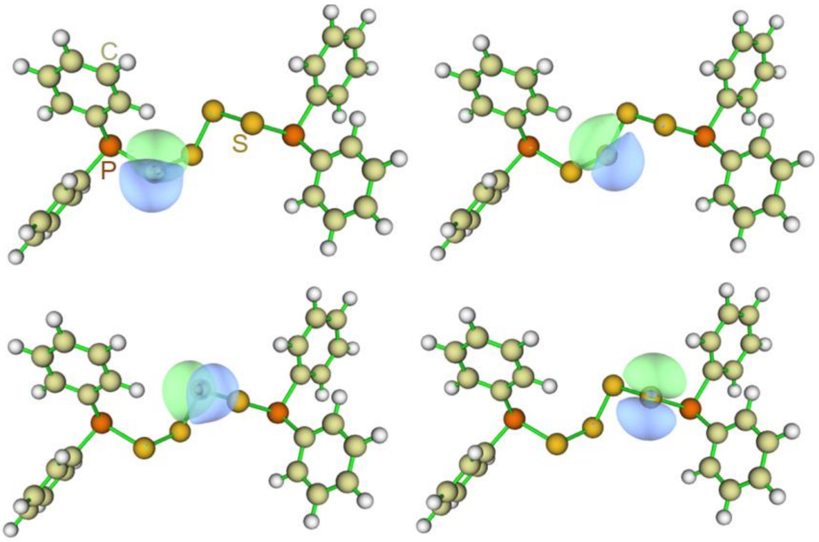

Supplementary Figure 23. Plots of the localized molecular orbitals (LMO) of the calculated BDPPTS and P, C, S are labeled.

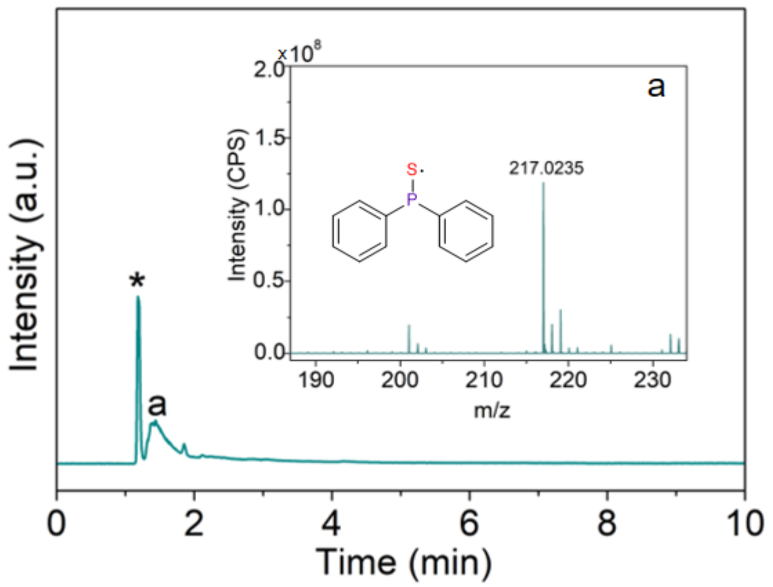

Supplementary Figure 24. TIC of the discharged product at 2.7 V and the insert MS shows the m/z of the ionized LiDPPT.

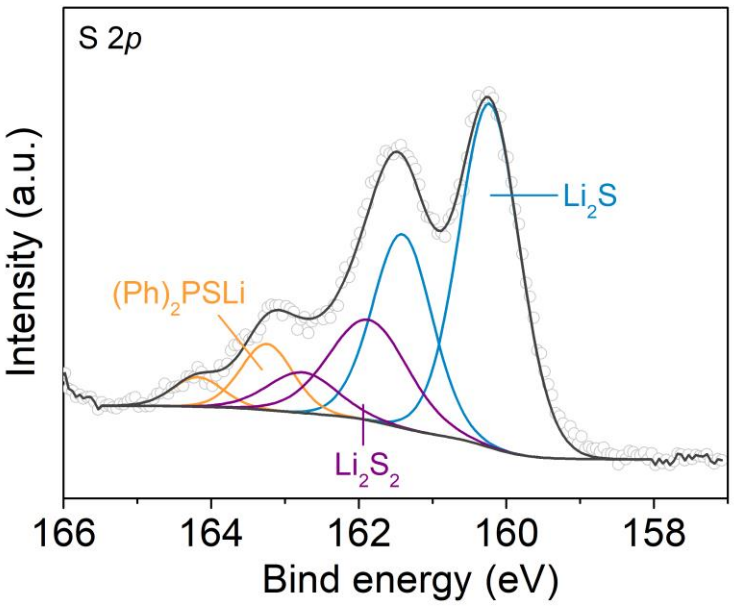

Supplementary Figure 25. XPS spectrum of S 2*p* of the BDPPTS electrode after discharge. The peak is mainly divided into three pairs of doublet peaks corresponding to Li_2S, Li_2S_2, and $(Ph)_2PSLi$.

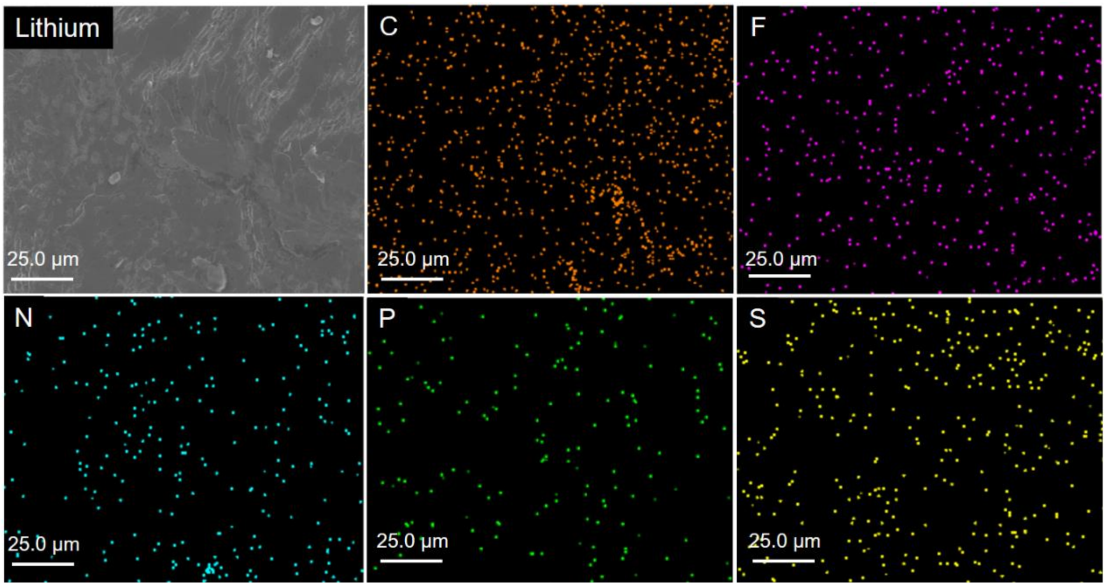

Supplementary Figure 26. SEM image and EDS elemental mapping of Li surface after 5 cycles at C/24 rate.

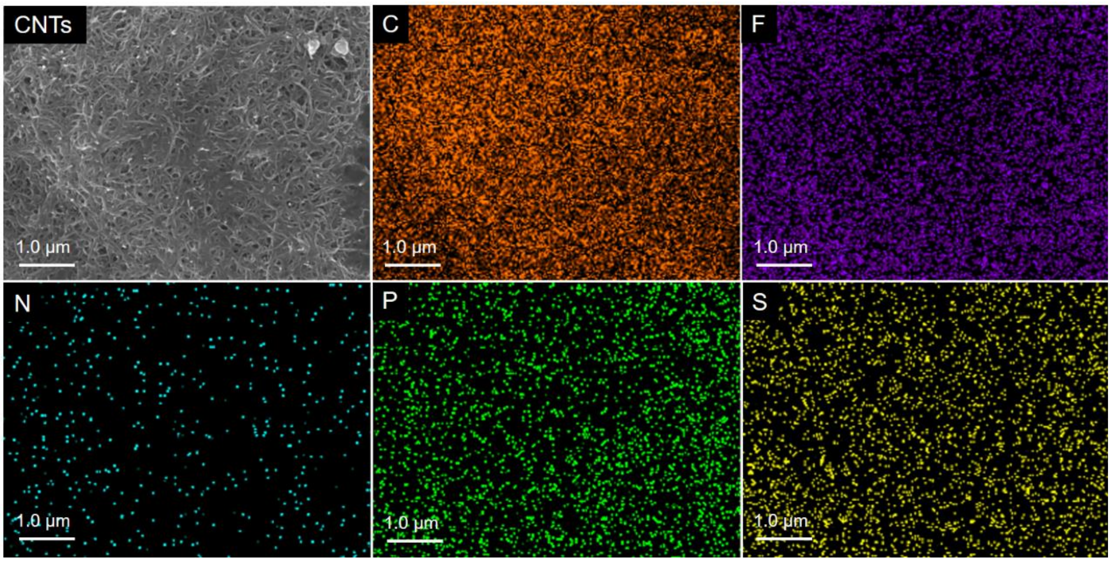

Supplementary Figure 27. SEM image and EDS elemental mapping of the cathode after 5 cycles at C/24 rate.

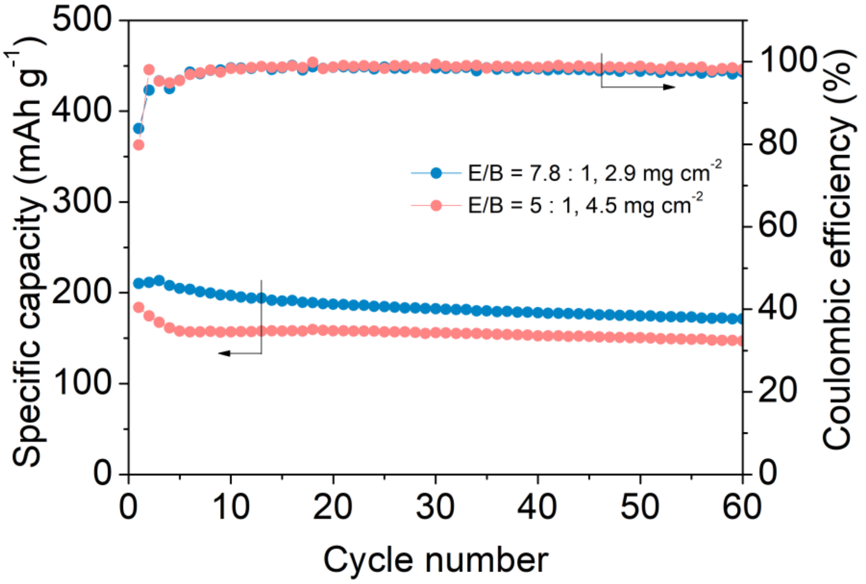

Supplementary Figure 28. Cycle performance of the Li/BDPPTS cell cycled at C/10 rate. The BDPPTS mass loading on the cathode are 2.9 and 4.5 mg cm^{-2} with the electrolyte/BDPPTS ratios of 7.8:1 and 5:1, respectively.

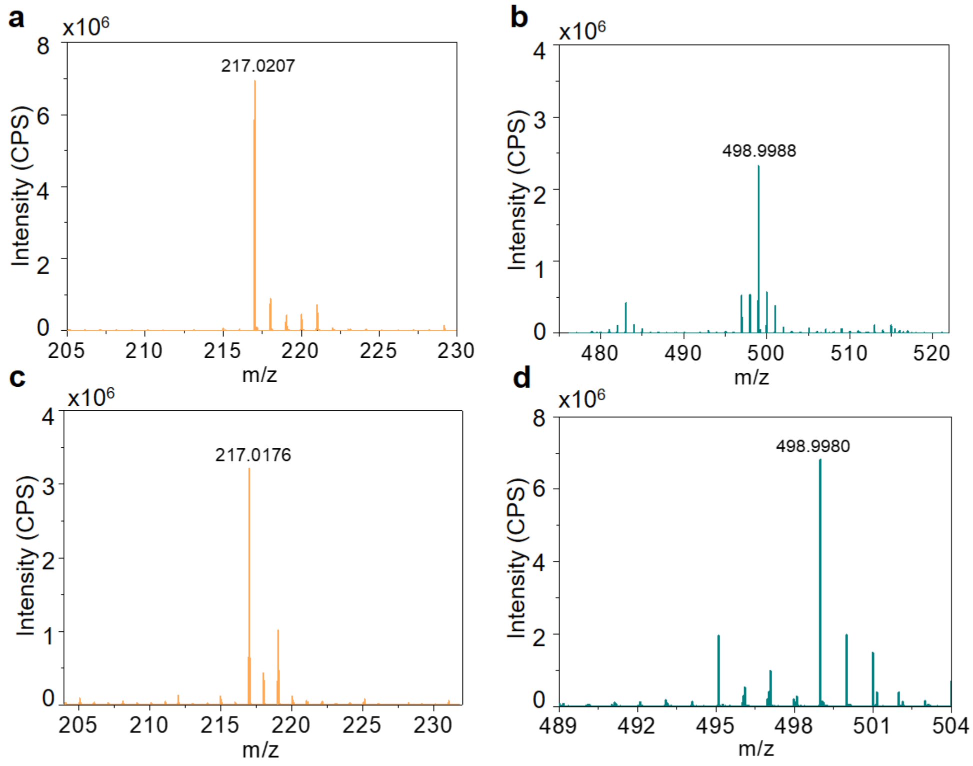

Supplementary Figure 29. The mass spectra of the discharged (a) and recharged (b) products after 10 cycles and the discharged (c) and recharged (d) products after 50 cycles in the voltage range of 2.5-3.4 V.

Supplementary Tables:

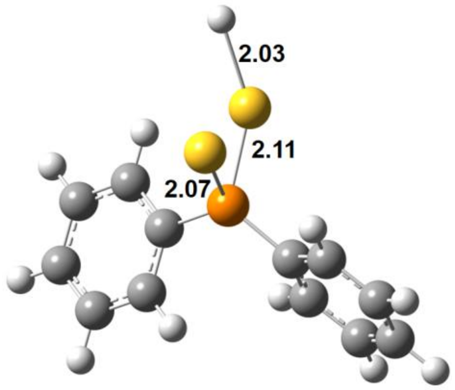

Supplementary Table 1. Coordinate of TS01 (619.47 *i*) optimized at the M062X/SMD/def2SVP level, the P=S, P-S and S-H bonds length were labeled (in Å).

Element	X	Y	Z
C	3.841004	-1.807145	-0.487165
C	2.645284	-2.505574	-0.381599
C	1.509215	-1.829995	0.091609
C	1.560248	-0.477968	0.469761
C	2.769679	0.202243	0.355594
C	3.902043	-0.458748	-0.121511
H	4.730657	-2.313245	-0.863629
H	2.590843	-3.55599	-0.672461
H	0.67773	0.040528	0.8511
H	2.824317	1.253155	0.641683
H	4.843775	0.084801	-0.212506
C	-1.531574	-1.821234	0.076803
C	-2.678149	-2.191245	0.798419
C	-1.570519	-0.808253	-0.897674
C	-3.869348	-1.522303	0.549575
H	-2.635195	-2.977826	1.553496
C	-2.775191	-0.152236	-1.130669
H	-0.680729	-0.527447	-1.465028
C	-3.916912	-0.506189	-0.409812
H	-4.764599	-1.785361	1.113979
H	-2.818744	0.643608	-1.87505
H	-4.854522	0.021001	-0.593533
S	0.022197	-4.73775	-0.260317
S	-0.045913	-4.136776	1.816267
H	0.726799	-6.59756	0.16447
P	-0.015463	-2.70471	0.316391

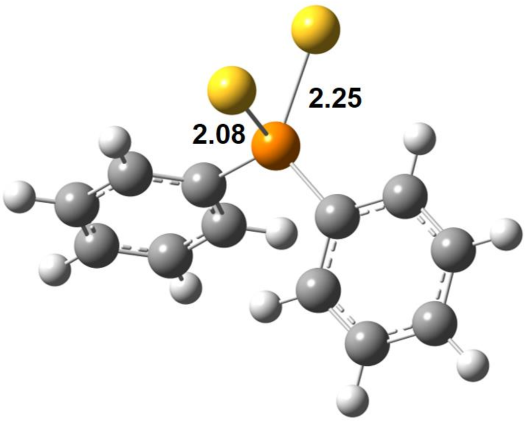

Supplementary Table 2. Coordinate of TS12 (1324.01 *i*) optimized at the M062X/SMD/def2SVP level, the P=S and P-S bonds length were labeled (in Å).

Element	X	Y	Z
**C**	2.423159	-2.625881	0.405834
**C**	1.288977	-1.820459	0.308684
**C**	1.418796	-0.486683	-0.103695
**C**	2.684731	0.033823	-0.410692
**C**	3.810554	-0.778205	-0.303647
**C**	3.682384	-2.108684	0.100856
**H**	2.319367	-3.662646	0.730234
**H**	0.310704	-2.231587	0.563615
**H**	2.781931	1.073177	-0.731445
**H**	4.793944	-0.369198	-0.54121
**H**	4.566874	-2.743065	0.17959
**C**	-1.547534	-0.342458	-0.098687
**C**	-2.538726	0.007296	0.825982
**C**	-1.769339	-1.392874	-1.006725
**C**	-3.733428	-0.712235	0.861991
**H**	-2.374511	0.837137	1.515986
**C**	-2.960933	-2.108302	-0.956675
**H**	-1.00756	-1.650806	-1.746711
**C**	-3.94505	-1.768706	-0.023018
**H**	-4.502589	-0.442191	1.587438
**H**	-3.12733	-2.929692	-1.655515
**H**	-4.882206	-2.327065	0.007336
**S**	0.063026	2.078767	1.220643
**S**	0.406599	2.673909	-1.01447
**P**	-0.016371	0.60525	-0.245198

Supplementary References

1 Shacklette, L. W., Toth, J. E., Murthy, N. S. & R. H. Baughman. Polyacetylene and polyphenylene as anode materials for nonaqueous secondary batteries. *J. Electrochem. Soc.* **132**, 1529-1535 (1985).

2 Suga, T., Ohshiro, H., Sugita, S., Oyaizu, K. & Nishide, H. Emerging N-type redox-active radical polymer for a totally organic polymer-based rechargeable battery. *Adv. Mater.* **21**, 1627-1630 (2009).

3 Jayaprakash, N., Shen, J., Moganty, S. S., Corona, A. & Archer, L. A. Porous hollow carbon@sulfur composites for high-power lithium-sulfur batteries. *Angew. Chem. Int. Ed.* **50**, 5904-5908 (2011).

4 Wu, M. *et al.* Highly Reversible Diphenyl trisulfide catholyte for rechargeable lithium batteries. *ACS Energy Lett.* **1**, 1221-1226 (2016).

5 Luo, C. *et al.* Azo Compounds derived from electrochemical reduction of nitro compounds for high performance Li-ion batteries. *Adv. Mater.* **30**, e1706498 (2018).

6 Bai, S. *et al.* Permselective metal-organic framework gel membrane enables long-life cycling of rechargeable organic batteries. *Nat. Nanotechnol.* **16**, 77-84 (2021).
